# GBCdb: RNA expression landscapes and ncRNA–mRNA interactions in gallbladder carcinoma

**DOI:** 10.1186/s12859-023-05133-2

**Published:** 2023-01-09

**Authors:** Li Guo, Yangyang Xiang, Yuyang Dou, Zibo Yin, Xinru Xu, Lihua Tang, Jiafeng Yu, Jun Wang, Tingming Liang

**Affiliations:** 1 Department of Bioinformatics, Smart Health Big Data Analysis and Location Services Engineering Lab of Jiangsu Province, School of Geographic and Biologic Information, Nanjing University of Posts and Telecommunications, Nanjing, 210023 China; 2 Jiangsu Key Laboratory for Molecular and Medical Biotechnology, School of Life Science, Nanjing Normal University, Nanjing, 210023 China; 3 Shandong Provincial Key Laboratory of Biophysics, Institute of Biophysics, Dezhou University, Dezhou, 253023 China

**Keywords:** Gallbladder carcinoma (GBC), Multi-omics, Expression landscape, RNA interaction

## Abstract

Gallbladder carcinoma (GBC), an aggressive malignant tumor of the biliary system, is characterized by high cellular heterogeneity and poor prognosis. Fewer data have been reported in GBC than other common cancer types. Multi-omics data will contribute to the understanding of the molecular mechanisms of cancer, cancer diagnosis and prognosis. Herein, to provide better understanding of the molecular events in GBC pathogenesis, we developed GBCdb (http://tmliang.cn/gbc/), a user-friendly interface for the query and browsing of GBC-associated genes and RNA interaction networks using published multi-omics data, which also included experimentally supported data from different molecular levels. GBCdb will help to elucidate the potential biological roles of different RNAs and allow for the exploration of RNA interactions in GBC. These resources will provide an opportunity for unraveling the potential molecular features of Gallbladder carcinoma.

**Supplementary Information:**

The online version contains supplementary material available at 10.1186/s12859-023-05133-2.

## Introduction

Gallbladder carcinoma (GBC), the most common cancer of the biliary tract [1, 2], has a dismal survival rate, which is largely caused by late diagnosis. Most patients with symptoms are found with incurable tumors, and the clinical outcome is very poor: the median survival time is less than 1 year and the 5-year overall survival rate is less than 5% [3]. Currently, the most effective treatment for GBC is surgery. However, because of asymptomatic characteristics at the early stage and the insidious onset and rapid progression of disease, few patients (less than 10%) are eligible for surgery [4]. Other treatments, such as chemotherapy, targeted therapy, and immune therapy, are available, but only a few patients have a promising prognosis. Therefore, early diagnosis of GBC is essential, and the identification of specific and sensitive biomarkers is critical to improve patient outcome.

Gene mutations and aberrant signaling pathways play key roles in GBC tumorigenesis. Mutations in TP53, ERBB2/ERBB3 and KRAS genes are frequently detected in GBC and are associated with clinical outcomes and treatment efficacy [5–8]. HER2 gene (ERBB2) amplification may be a low-frequency driver with potential predictive value [9]. RIP-1 inhibits the ability of GBC cells to grow and invade in vitro [10], and p53 gene expression is a prognostic factor for subserosal GBC [11]. Non-coding RNAs (ncRNAs), mainly including microRNAs (miRNAs), long non-coding RNAs (lncRNAs) and circular RNAs (circRNAs), can function as important regulators in gene expression. Many ncRNAs have critical roles in tumorigenesis, and some ncRNAs function as competing endogenous RNAs (ceRNAs) to perturb gene expression. For example, miR-365 inhibits the progression of GBC by directly targeting RAC1 and may be a novel prognosis biomarker for GBC [12]. The lncRNA TMPO-AS1 promotes cell proliferation, migration, invasion and epithelial-to-mesenchymal transition by regulating the miR-1179/E2F2 axis [13]. miR-4733-5p promotes GBC progression by directly targeting kruppel like factor 7 [14], and miR-4461 may inhibit the progression of GBC by regulating EGFR/AKT signaling [15]. The mechanisms underlying the occurrence and development of GBC may involve aberrant alterations of multiple molecular pathways. Thus, multi-omics analysis of the molecular landscape of GBC is critical to understand the pathogenesis of this complex disease.

**Fig. 1:**
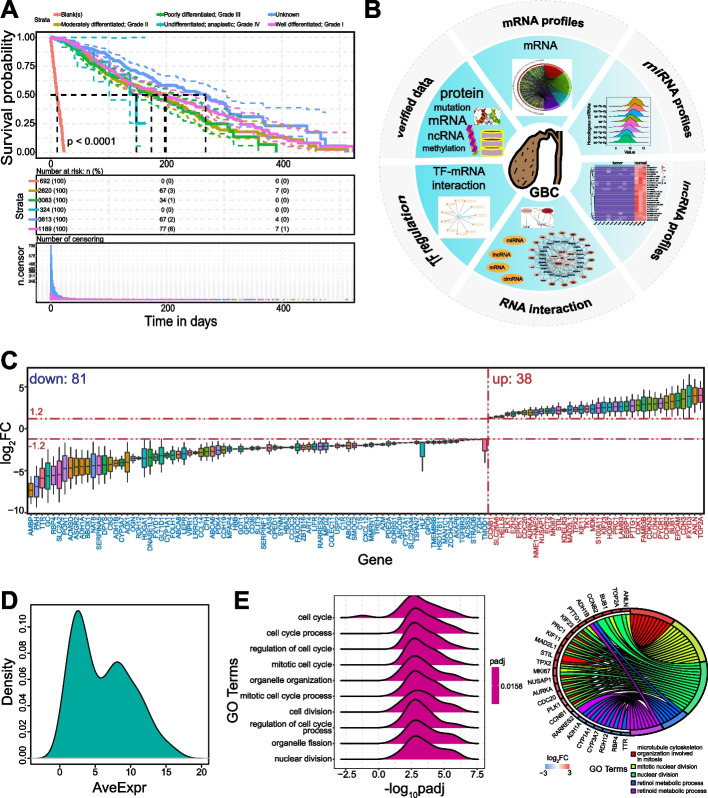
The main components of GBCdb and mRNA expression profiles. **A** Survival analysis on the basis of information from the SEER database. Significant differences were detected among different grades. **B** The main components GBCdb, including mRNA expression profiles, ncRNA expression profiles (including miRNA, lncRNA and circRNA), RNA interaction networks and TF regulation networks and experimentally supported GBC-associated data. **C** The distributions of log_2_FC values of screened differentially expressed genes (119 genes). These genes exhibited abnormal expression in at least two datasets. **D** The distribution of AveExpr (average expression of all average-values) of the screened 119 differentially expressed genes. **E** Significantly enriched Gene Ontology (GO) terms using clusterProfiler. The right panel shows enriched GO terms from the GBC-associated gene expression patterns using GO plot

The molecular events underlying GBC pathogenesis, especially from multi-omics levels, are still unclear. Because of the highly aggressive nature and poor prognosis of this cancer, and with the significant differences among different grades (Fig. 1A), it poses a great challenge to relevant studies. To address these limitations and understand the molecular landscapes from the multiple levels, we constructed GBCdb (http://tmliang.cn/gbc) to exhibit the GBC-associated RNA expression landscape and potential RNA interactions (among mRNAs, miRNAs, lncRNAs and circRNAs), along with experimentally verified GBC-associated genes obtained from the literatures (Fig. 1B). GBCdb is a user-friendly database for browsing, searching, and downloading of GBC-related multi-omics results, especially RNA interaction networks that can provide potential ncRNA relationships in the gene expression process. Our findings provide a platform to improve understanding of the detailed multi-omics RNA landscapes of GBC, especially for GBC-associated RNA interactions, which will support future studies on cancer treatment.

## Results

### The overall GBC-associated RNA expression profiles

mRNA expression data were collected from public data (Additional file 1: Table S1). Many genes showed consistent expression patterns among different datasets (Fig. 1C), and most exhibited dominant expression patterns (Fig. 1D). A total of 119 differentially expressed mRNAs were detected in at least two datasets. Gene Ontology analysis indicated that these candidate GBC-associated genes have potential roles in nuclear division and cell cycle pathways (Fig. 1E). Because of the small sample sizes and limited datasets, all differentially expressed mRNAs were used in subsequent analyses.

**Fig. 2:**
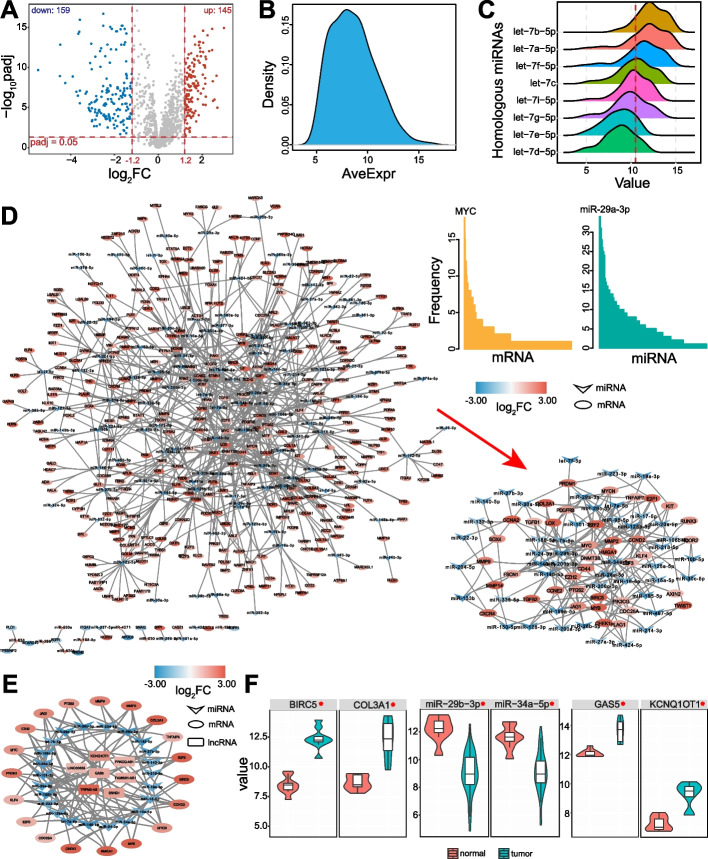
RNA interaction network. **A** A volcano plot shows the differentially expressed miRNAs identified from GSE104165. **B** The distribution of AveExpr of the screened differentially expressed miRNAs from **A**. **C** Distribution of expressions of homologous miRNAs in the let-7 gene family in tumor samples. The red dotted line indicates the median value of the total expression levels. **D** The miRNA-mRNA interaction network of the differentially expressed RNAs (at least in one dataset). A specific network is presented with multiple interactions for some miRNAs and mRNAs. The detailed distributions of frequencies are also presented. **E** An example of mRNA-miRNA-lncRNA network; each RNA is significantly up-regulated or down-regulated in tumor samples. **F** Examples of several mRNAs, miRNAs and lncRNAs that are significantly dysregulated in GBC. *Indicates |log_2_FC|> 1.2 and padj < 0.05

To understand the potential RNA interactions among different RNAs, especially the regulatory roles of ncRNAs, expression analysis was performed to screen GBC-associated ncRNAs. Some differentially expressed miRNAs were identified (Fig. 2A, B), and 304 miRNAs were detected in GSE104165 [16]. Some homologous miRNAs, such as those in the let-7 gene family, showed similar expression patterns (Fig. 2C), indicating that they may exhibit similar functions via homologous sequence. From the experimentally validated miRNA-mRNA interactions and expression patterns, a complex candidate miRNA-mRNA interaction network was constructed (Fig. 2D). Most involved mRNAs showed significantly up-regulated expression and their negative regulators showed down-regulated expression. Some miRNAs had multiple target mRNAs, especially miR-29a-3p, indicating this miRNA has multiple regulatory roles in gene expression. From the miRNA-lncRNA interactions, the RNA network was further constructed using three different RNAs that presented potential regulatory relationships based on ceRNA network (Fig. 2E). lncRNAs may act as miRNA sponges to perturb mRNA expression. All these RNAs showed abnormal expression patterns in tumor samples (Fig. 2F), implicating the complex interactions among different RNAs. Potential relationships were detected among miRNAs, mRNAs and circRNAs, but no significantly dysregulated circRNAs were obtained due to the limited circRNA data.

**Fig. 3:**
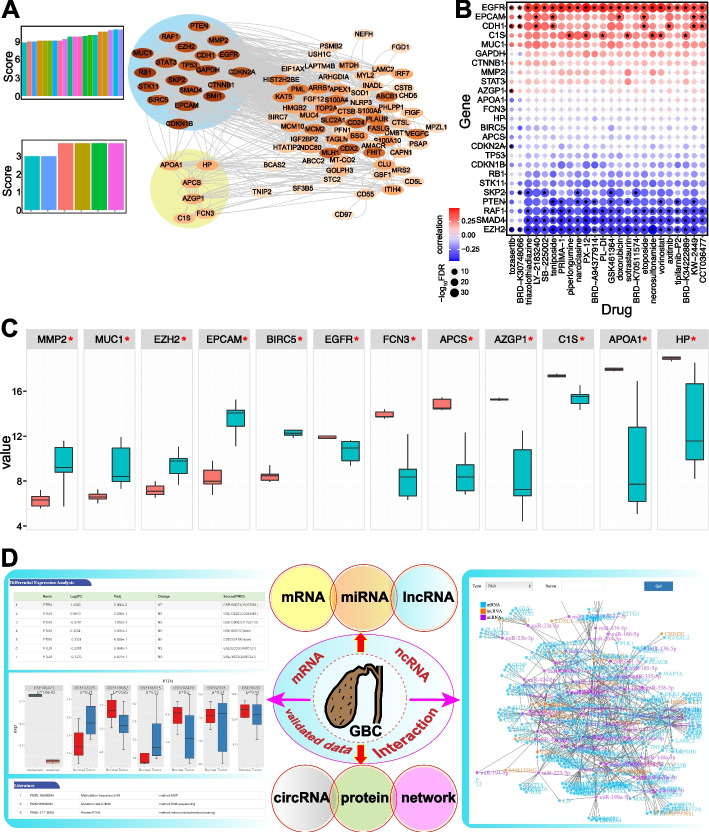
A primary analysis of experimentally supported GBC-associated proteins and overview of GBCdb. **A** PPI network of the screened experimentally supported GBC-associated proteins. Two clusters were identified, and the detailed score distributions are presented. **B** The correlations of genes and drugs (top of 24 drugs) based on Cancer Cell Line Encyclopedia (CCLE). *Indicates significant correlations (|correlation coefficient|> 0.3 and FDR < 0.05). **C** Expressions of significantly differentially expressed genes in GSE76633 (|log_2_FC|> 1.2 and padj < 0.05). **D** Overview of GBCdb. The left panel shows an example of a searched mRNA, and the right panel shows a total mRNA-miRNA-lncRNA network. All these RNAs are significantly dysregulated. In each miRNA-mRNA or miRNA-lncRNA pair, one is significantly up-regulated while the other is significantly down-regulated in tumor samples. RNA expression patterns can be queried in different datasets and corresponding experimentally supported data are also provided in different molecular levels

We performed further analysis to screen candidate hub genes from experimentally supported GBC-associated proteins. A total of 26 candidate hub genes were identified (Fig. 3A), including RB1, MUC1, SKP2, HP, APCS and AZGP1 genes. MUC1 has been associated with the progression of GBC [17], and Skp2 may be an independent prognostic factor for GBC [18]; these studies suggested the potential roles of these factors in the occurrence and development of cancer. These genes were also potential drug targets (Fig. 3B), and most exhibited significantly dysregulated expression patterns in some datasets (such as in GSE76633) (Fig. 3C). Experimentally supported GBC-related genes, proteins and ncRNAs and the mutation or methylation patterns represent a basis for further study for this complex disease.

### Web interface

Based on the collected data and primary analysis, GBCdb was developed to present information on the molecular events in GBC pathogenesis. GBCdb contained results from the primary analysis of expression profiles of RNAs, including mRNA, miRNA, lncRNA and circRNA, RNA interaction networks, TF regulatory networks and experimentally supported GBC-associated genes. GBCdb has a user-friendly web interface (Fig. 3D), which allows users to query the database via multiple modules. (1) The “Search” module can be used to search different RNA types, including mRNA, miRNA, lncRNA and circRNA, and the detailed expression patterns in different datasets are presented to explore expression patterns. Because of the limited GBC-related data, to obtain more data, additional experimentally supported data for a specific gene (such as mutation or methylation data) are also presented. For lncRNA, the significant drug-lncRNA correlations are also presented. While no significantly dysregulated circRNAs were detected because of the limited data, miRNA-circRNA interactions are also presented to provide potential insights of the role of circRNAs as miRNA sponges. (2) The “RNA network” module presents the total originally screened candidate mRNA-miRNA-lncRNA network. For each miRNA-mRNA or miRNA-lncRNA pair, potential expression relationships are first screened. LncRNAs can act as miRNA sponges to perturb mRNA expression, and the RNA interaction network provides a complex regulatory network. The detailed interactions for each gene are presented to indicate the potential regulatory relationships. The TF-regulatory network is also presented for the differentially expressed genes. Users can select the type of input molecule (RNA or TF-target) using the pull-down menu and then enter the name in the search box. Fuzzy search and input prompt are supported here. Users can quickly obtain the upstream and downstream genes or transcription regulators of the target molecule. (3) The “Download” module is used to download all the relevant differential expression profiles in different datasets. (4) The “Help” module contains detailed documentation and tutorials. GBCdb welcomes any feedback via the email address provided on the “Contact Us” page.

## Discussion and future prospects

GBC, an extremely malignant tumor, has high invasion and metastasis rates and is characterized with poor prognosis and a high mortality rate. The precise molecular mechanisms of GBC remain unclear. Although many studies have reported critical GBC-associated genes, few studies have focused on the expression landscapes via integrative analysis of different RNAs. Many genes and molecules, including diverse ncRNAs, play critical roles in the pathophysiological process, and better understanding of their interactions is important to understand the molecular mechanisms of GBC. Single-cell RNA sequencing allows for the exploration of intratumoral heterogeneity and cancer progression [19] and insights into the occurrence and development of cancer. In this study, we aimed to provide detailed molecular expression profiles and GBC-associated RNA interaction networks that will help contribute to a better understanding of cancer from multi-omics data. We collected and analyzed relevant data from public databases and literatures, and then developed GBCdb, a database containing multi-omics data and RNA interaction networks. Multi-omics data were mainly obtained from GEO database (Additional file 1: Table S1), including expression profiles of mRNAs, miRNAs, lncRNAs and circRNAs. Other relevant experimentally supported data were obtained from published studies, mainly including GBC-associated genes and molecular features. The detailed expression patterns and the potential RNA interactions among diverse RNAs helped establish GBC-associated RNA landscapes, which were used for screening candidate critical RNAs. Although these RNA interactions were primarily obtained from different datasets due to limited GBC-related data, the candidate RNA networks still provided the potential interactions or cross-talks among different RNAs, even among different biological pathways.

In the future, GBCdb will contain more data from multiple molecular levels. The current data are not sufficient for a systematic analysis because of the limited datasets, which is partly because of the poor prognosis of GBC. The experimentally validated RNA interactions will be contained to construct RNA interaction network to track the coding-non-coding RNA regulatory network, especially on the basis of the ceRNA network. We will update GBCdb by collecting and reanalyzing single-cell sequencing data. Finally, using screened GBC-associated genes, a pan-cancer analysis will be performed to understand the potential expression patterns and RNA interaction networks in different cancer types, which will contribute to further understanding of the biological roles in GBC.

Taken together, GBCdb might provide a useful resource for understanding the detailed expression landscapes, RNA interaction networks among different RNAs and experimentally supported data from published studies. GBCdb provides a user-friendly interface for the query and browsing of detailed information and will help understand the potential RNA interactions and biological functions associated with GBC. The database will be updated as more multi-omics data are available. We believe that GBCdb will be a valuable resource for understanding the RNA expression landscapes and interaction networks that will contribute to exploring the potential molecular mechanisms of GBC.

## Materials and methods

### Data collection

GBC-related multi-omics data, mainly including expression data of mRNA, miRNA, lncRNA and circRNA, were retrieved from the NCBI GEO database [16, 20–24] (Additional file 1: Table S1). In order to further understand GBC-associated genes, relevant experimentally supported data were also collected from published studies. For interactions between different RNAs, particularly ncRNA–mRNA interactions, experimentally supported miRNA-mRNA interactions and miRNA-lncRNA interactions were obtained from starBase 2.0 [25], and miRNA-circRNA interactions were mainly downloaded from circbank [26]. The drug-lncRNA correlations were obtained from lncMAP to present the potential roles of lncRNA in cancer treatment [27]. TF-target data were downloaded from hTFtarget to explore the TF-regulatory network [28].

### Differential RNA expression profiles and function enrichment analysis

For the obtained RNA dataset, differentially expressed RNA profiles were estimated with limma package [29]. To reduce the impacts of batch effects, we used the ComBat [30] in the process. A dysregulated RNA was defined if |log_2_FC|> 1.2 and padj < 0.05. Functional analysis of candidate genes was performed with The Database for Annotation, Visualization and Integrated Discovery (DAVID) version 6.8 [31] and clusterProfiler 4.0 [32] to understand their potential biological roles. Additionally, to estimate the potential correlations of candidate hub genes and drugs, drug sensitivity analysis was performed using GSCA [33].

### Survival analysis

To evaluate whether cancer grades had potential prognostic values in GBC patients, survival analysis was performed using the Surveillance, Epidemiology, and End Results (SEER) dataset using survival R package. All cases were obtained from the SEER Program (http://www.seer.cancer.gov) SEER*Stat database released in May 2022: version 8.4.1. The log-rank test was used to calculate the differences among the difference grades. *p* < 0.05 indicated statistical significance.

### Network visualization and statistical analysis

From the potential interactions among different RNAs, an RNA interaction network was constructed using Cytoscape 3.8.2 [34]. The collected experimentally supported GBC-associated proteins were used to survey the potential hub genes via protein–protein interaction (PPI) networks using the STRING online database [35]. The Wilcoxon rank-sum test was used to validate the potential differences between different groups. All analyses were performed with R programming language (version 4.0.5).

Using the collected data and primary analysis, we developed GBCdb to query and browse GBC-associated RNA expression profiles, RNA interactions and experimentally supported multi-omics data, TF-regulatory network, and etc.

## Supplementary Information

**Additional file 1**. GBC related datasets involved in this study.

## Data Availability

GBCdb (http://tmliang.cn/gbc/) is freely available to the public without registration or login requirements.

## References

[1] Varshney S, Butturini G, Gupta R (2002). Incidental carcinoma of the gallbladder. Eur J Surg Oncol.

[2] Wistuba II, Gazdar AF (2004). Gallbladder cancer: lessons from a rare tumour. Nat Rev Cancer.

[3] Mao W, Deng F, Wang D, Gao L, Shi X (2020). Treatment of advanced gallbladder cancer: a SEER-based study. Cancer Med.

[4] Hundal R, Shaffer EA (2014). Gallbladder cancer: epidemiology and outcome. Clin Epidemiol.

[5] Li M, Liu F, Zhang F, Zhou W, Jiang X, Yang Y (2019). Genomic ERBB2/ERBB3 mutations promote PD-L1-mediated immune escape in gallbladder cancer: a whole-exome sequencing analysis. Gut.

[6] Li M, Zhang Z, Li X, Ye J, Wu X, Tan Z (2014). Whole-exome and targeted gene sequencing of gallbladder carcinoma identifies recurrent mutations in the ErbB pathway. Nat Genet.

[7] Moreno M, Pimentel F, Gazdar AF, Wistuba II, Miquel JF (2005). TP53 abnormalities are frequent and early events in the sequential pathogenesis of gallbladder carcinoma. Ann Hepatol.

[8] Shukla SK, Singh G, Shahi KS, Bhuvan, Pant P. Genetic Changes of P(53) and Kras in Gallbladder Carcinoma in Kumaon Region of Uttarakhand. J Gastrointest Cancer. 2020;51:552–9. 10.1007/s12029-019-00283-0 31396884

[9] Albrecht T, Rausch M, Roessler S, Geissler V, Albrecht M, Halske C (2020). HER2 gene (ERBB2) amplification is a low-frequency driver with potential predictive value in gallbladder carcinoma. Virchows Arch.

[10] Zhu G, Chen X, Wang X, Li X, Du Q, Hong H (2014). Expression of the RIP-1 gene and its role in growth and invasion of human gallbladder carcinoma. Cell Physiol Biochem.

[11] Roa EI, Lantadilla HS, Ibacache SG (2009). de Aretxabala UX [p53 and p27 gene expression in subserosal gallbladder carcinoma]. Rev Med Chil.

[12] Jiang ZB, Ma BQ, Feng Z, Liu SG, Gao P, Yan HT (2021). miR-365 inhibits the progression of gallbladder carcinoma and predicts the prognosis of Gallbladder carcinoma patients. Cell Cycle.

[13] Sui Z, Sui X (2021). Long non-coding RNA TMPO-AS1 promotes cell proliferation, migration, invasion and epithelial-to-mesenchymal transition in gallbladder carcinoma by regulating the microRNA-1179/E2F2 axis. Oncol Lett.

[14] Hu X, Zhang J, Bu J, Yang K, Xu S, Pan M (2022). MiR-4733-5p promotes gallbladder carcinoma progression via directly targeting kruppel like factor 7. Bioengineered.

[15] Yan X, Yang P, Liu H, Zhao Y, Wu Z, Zhang B (2022). miR-4461 inhibits the progression of Gallbladder carcinoma via regulating EGFR/AKT signaling. Cell Cycle.

[16] Goeppert B, Truckenmueller F, Ori A, Fritz V, Albrecht T, Fraas A (2019). Profiling of gallbladder carcinoma reveals distinct miRNA profiles and activation of STAT1 by the tumor suppressive miRNA-145-5p. Sci Rep.

[17] Park SY, Roh SJ, Kim YN, Kim SZ, Park HS, Jang KY (2009). Expression of MUC1, MUC2, MUC5AC and MUC6 in cholangiocarcinoma: prognostic impact. Oncol Rep.

[18] Li SH, Li CF, Sung MT, Eng HL, Hsiung CY, Huang WW (2007). Skp2 is an independent prognosticator of gallbladder carcinoma among p27(Kip1)-interacting cell cycle regulators: an immunohistochemical study of 62 cases by tissue microarray. Mod Pathol.

[19] Chen P, Wang Y, Li J, Bo X, Wang J, Nan L (2021). Diversity and intratumoral heterogeneity in human gallbladder cancer progression revealed by single-cell RNA sequencing. Clin Transl Med.

[20] Qin Y, Zheng Y, Huang C, Li Y, Gu M, Wu Q (2021). Downregulation of miR-181b-5p inhibits the viability, migration, and glycolysis of gallbladder cancer by upregulating PDHX under hypoxia. Front Oncol.

[21] Li H, Hu Y, Jin Y, Zhu Y, Hao Y, Liu F (2020). Long noncoding RNA lncGALM increases risk of liver metastasis in gallbladder cancer through facilitating N-cadherin and IL-1beta-dependent liver arrest and tumor extravasation. Clin Transl Med.

[22] Wu XS, Wang F, Li HF, Hu YP, Jiang L, Zhang F (2017). LncRNA-PAGBC acts as a microRNA sponge and promotes gallbladder tumorigenesis. EMBO Rep.

[23] Xu S, Zhan M, Jiang C, He M, Yang L, Shen H (2019). Genome-wide CRISPR screen identifies ELP5 as a determinant of gemcitabine sensitivity in gallbladder cancer. Nat Commun.

[24] Wang S, Zhang Y, Cai Q, Ma M, Jin LY, Weng M (2019). Circular RNA FOXP1 promotes tumor progression and Warburg effect in gallbladder cancer by regulating PKLR expression. Mol Cancer.

[25] Li JH, Liu S, Zhou H, Qu LH, Yang JH. starBase v2.0: decoding miRNA-ceRNA, miRNA-ncRNA and protein-RNA interaction networks from large-scale CLIP-Seq data. Nucleic Acids Res. 2014;42:D92–7. 10.1093/nar/gkt1248 PMC3964941 24297251

[26] Liu M, Wang Q, Shen J, Yang BB, Ding X (2019). Circbank: a comprehensive database for circRNA with standard nomenclature. RNA Biol.

[27] Li Y, Li L, Wang Z, Pan T, Sahni N, Jin X (2018). LncMAP: Pan-cancer atlas of long noncoding RNA-mediated transcriptional network perturbations. Nucleic Acids Res.

[28] Zhang Q, Liu W, Zhang HM, Xie GY, Miao YR, Xia M (2020). hTFtarget: a comprehensive database for regulations of human transcription factors and their targets. Genom Proteom Bioinf.

[29] Ritchie ME, Phipson B, Wu D, Hu Y, Law CW, Shi W (2015). limma powers differential expression analyses for RNA-sequencing and microarray studies. Nucleic Acids Res.

[30] Leek JT, Johnson WE, Parker HS, Jaffe AE, Storey JD (2012). The sva package for removing batch effects and other unwanted variation in high-throughput experiments. Bioinformatics.

[31] Huang DW, Sherman BT, Lempicki RA (2009). Systematic and integrative analysis of large gene lists using DAVID bioinformatics resources. Nat Protoc.

[32] Wu T, Hu E, Xu S, Chen M, Guo P, Dai Z (2021). clusterProfiler 4.0: a universal enrichment tool for interpreting omics data. Innovation (Camb)..

[33] Liu CJ, Hu FF, Xia MX, Han L, Zhang Q, Guo AY (2018). GSCALite: a web server for gene set cancer analysis. Bioinformatics.

[34] Shannon P, Markiel A, Ozier O, Baliga NS, Wang JT, Ramage D (2003). Cytoscape: a software environment for integrated models of biomolecular interaction networks. Genome Res.

[35] Szklarczyk D, Gable AL, Lyon D, Junge A, Wyder S, Huerta-Cepas J (2019). STRING v11: protein–protein association networks with increased coverage, supporting functional discovery in genome-wide experimental datasets. Nucleic Acids Res.

