# Supplementary material for: GBCdb: RNA expression landscapes and ncRNA–mRNA interactions in gallbladder carcinoma
Source: BMC Bioinformatics. 2023 Jan 9;24:12. doi: 10.1186/s12859-023-05133-2 (PMC9830852; doi:10.1186/s12859-023-05133-2)
Supplement: Supplementary file 1 — Additional file 1. GBC related datasets involved in this study. [file 12859_2023_5133_MOESM1_ESM.docx]

**Supplementary files**

**Table S1. The obtained GBC-related GEO data.**

| **No.** | **Level** | **Number (tumor)** | **Number (normal)** | **Subtypes** | **PMID** |
| --- | --- | --- | --- | --- | --- |
| GSE62335 | lncRNA | 5 | 5 | Not applicable | 34485121 |
| GSE106671 | lncRNA | 3(parental) | 3(metastatic) | Not applicable | 33252861 |
| GSE202479 | lncRNA | 3 | 10 | Adenocarcinoma | None |
| GSE132223 | lncRNA | 3 | 6 | Not applicable | 33252861 |
| GSE74048 | lncRNA | 3 | 3 | Not applicable | None |
| GSE166915 | lncRNA | 5 | 5 | Not applicable | None |
| GSE76633 | lncRNA | 9 | 9 | Adenocarcinoma | 28887321 |
| GSE90001 | miRNA | 4 | 4 | Adenocarcinoma | 28887321 |
| GSE104165 | miRNA | 40 | 8 | Not applicable | 30886199 |
| GSE106671 | mRNA | 3(parental) | 3(metastatic) | Not applicable | 33252861 |
| GSE202479 | mRNA | 3 | 10 | Adenocarcinoma | None |
| GSE139682 | mRNA | 10 | 10 | Not applicable | 31792210 |
| GSE166915 | mRNA | 5 | 5 | Not applicable | None |
| GSE76633 | mRNA | 9 | 9 | Adenocarcinoma | 28887321 |
| GSE100363 | circRNA | 4 | 4 | Not applicable | 31623628 |
| GSE166915 | circRNA | 5 | 5 | Not applicable | None |

Not applicable: No subtype information is provided in the dataset.
